# Parental emotions in families of children and adolescents with and without autism spectrum disorder

**DOI:** 10.1590/1807-3107bor-2024.vol38.0106

**Published:** 2024-11-08

**Authors:** Gustavo Correia Basto da Silva, Ramon Targino Firmino, Ana Beatriz Dantas Nogueira, Andreza Cristina de Lima Targino Massoni, Sérgio D’Ávila

**Affiliations:** (a) Universidade Estadual da Paraíba – UEPB, Postgraduate Program in Dentistry, Campina Grande, PB, Brazil.; (b) Universidade Federal de Campina Grande – UGCG, Academic Unit of Biological Sciences, Patos, PB, Brazil.; (c) Universidade Estadual da Paraíba – UEPB, Department of Dentistry, Campina Grande, PB, Brazil.

**Keywords:** Autism Spectrum Disorder, Mental Health, Caregivers, Emotions

## Abstract

This study assessed the influence of sociodemographic conditions, oral hygiene habits, and the socio-psychological need for orthodontic treatment on the emotions of caregivers of children with and without autism spectrum disorder (ASD). We conducted a comparative cross-sectional study with families of individuals aged 6 to 14 years at a reference center for neurodevelopmental disorders and two dental school clinics in northeastern Brazil. Caregiver emotions were assessed using the ‘Parental Emotions’ domain of the Family Impact Scale (FIS). We analyzed sociodemographic variables, oral hygiene habits, and sociopsychological need for orthodontic treatment using the esthetic component of the Index of Orthodontic Treatment Need (IOTN). We used descriptive and hierarchical Poisson regression analyses with robust variance (α = 5%). The study included 144 families evenly distributed across the groups. The caregiver group with ASD demonstrated a higher total score for parental emotions (p < 0.001). Factors associated with this factor included caregiver responsible for brushing (PR = 1.34; 95%CI: 1.12–1.59), mandatory need for orthodontic treatment (PR = 1.25; 95%CI: 1.07–1.46), and caregivers’ education up to 8 years (PR = 1.45; 95%CI: 1.02–2.07). Caregivers with lower income showed a lower prevalence of parental emotions (PR = 0.57; 95%CI: 0.35–0.93). Caregivers of children with ASD exhibited a higher emotional burden. Factors associated with parental emotions included responsibility for tooth brushing attributed to caregivers, sociopsychological need for orthodontic treatment, and family income.

## Introduction

Autism spectrum disorder (ASD) encompasses a variety of conditions characterized by a certain degree of difficulty with social interaction and communication.^1^ It is estimated that 1 in every 100 children worldwide has autism, underscoring its relevance to public health.^2^ Some individuals with autism may live independently, while others require support. This population may also experience sensory hypersensitivity, hyperfocus, and difficulty recognizing emotions or physical sensations.^3^ When present, these conditions affect the autonomy of these individuals, who constantly rely on support to carry out daily activities, including oral hygiene, with parents and/or caregivers being the main providers of this assistance.^4^

Caregivers of children with ASD face higher incidence of stress, anxiety, and depression compared to those caring for neurotypical children and those with other disabilities, impacting family relationships.^5^ Another study identified that parents of individuals with ASD exhibited moderate levels of daily overload, as well as negative life changes and borderline symptoms of depression and anxiety.^6^ Parental burden is influenced by disruptive behaviors of individuals with ASD, financial issues, changes in social relationships, physical health problems, lack of social support, alterations in daily routine, and low ability to cope with stress.^6^

A study in a Brazilian sample sought to determine the prevalence of anxiety and depression symptoms and to analyze the quality of life (QoL) of adults during the pandemic across four groups: "childless", "children without mental problems", "children with autism", and "children with other mental problems". The results revealed that anxiety and depression symptoms were higher and QoL scores were lower in parents of children with autism.^7^ In contrast, a study conducted in Qatar did not identify differences in QoL domains between groups of caregivers of children with and without ASD.^8^ Despite ample evidence indicating that these families face significant stress, knowledge gaps and conflicting results persist.^9^

QoL reflects the overall well-being of the individual and is an essential construct in investigating developmental disabilities, potentially encompassing parental emoticons.^10^ Assessing the impact of oral and sociodemographic conditions on family dynamics is important for understanding the needs and challenges faced by caregivers of children and adolescents with ASD, guiding public policies and health programs.^11^ Evidence indicates that parental mental health significantly impacts the development and well-being of children with disabilities.^12^ A study on the effect of dental anesthesia on Chinese parents found that their emotions influenced their children's reactions, increasing muscle tension, tachycardia, hyperventilation, and sensitivity to pain.^13^

A previous study has examined the socio-psychological necessity of orthodontic treatment in individuals with ASD,^14^ yet there are no reported studies assessing its impact on parents/caregivers. The esthetic component of the Index of Orthodontic Treatment Need (IOTN) assesses this variable.^15^ Investigating how the esthetic burden of malocclusion impacts the emotions of parents/caregivers is crucial to understanding the factors that drive parents to seek orthodontic consultation, as the esthetic aspect plays a significant role in the decision-making process.^14^ Due to the relevance of the topic and the scarcity of studies on the subject, this type of research also contributes to gather evidence on the psychosocial impact of ASD.^13,16^

Thus, the present study aimed to assess the influence of sociodemographic conditions, oral hygiene habits, and socio-psychological treatment need on the emotions of caregivers of families with children and adolescents with and without ASD.

## Methodology

### Ethical aspects

The study rigorously adhered to ethical guidelines for research involving human subjects, as outlined in the Helsinki Declaration. We reported this study in accordance with the Strengthening the Reporting of Observational Studies in Epidemiology (STROBE) guidelines. The project obtained approval from the Brazil Research Ethics Committee, as documented in Approval No. 4.209.751. Informed consent, including the use of image rights, was obtained from all caregivers involved in the study.

### Study design

We conducted a comparative cross-sectional study with children and adolescents with and without ASD and their respective caregivers in Campina Grande, Paraíba, Brazil.

### Context

Our study was conducted from December 2021 to October 2022 at a specialized rehabilitation center catering to patients with neurodevelopmental disorders, and families for the control group were recruited in dental clinics associated with two dental schools. Prior to data collection, meetings were held with institution managers to outline study objectives and methodologies, and to obtain institutional authorization and family lists. Two groups were formed: one comprising children and adolescents with ASD and their caregivers and another comprising families without ASD.

### Sample calculation

Participants were recruited through non-probabilistic sampling due to COVID-19 restrictions. The calculation of the final sample size was conducted using the G*Power program (Franz Faul, University of Kiel, Kiel, Germany), version 3.1, based on a significance level of 5%, effect size of 0.70, and test power of 0.95. A minimum sample of 55 participants per group was established, with an additional 20% attrition margin to compensate for potential sample losses, resulting in a final minimum sample size of 66 participant pairs per group to ensure statistical power and reliability.

### Training and calibration

An orthodontic specialist, calibrated in two stages by an experienced researcher, conducted the data collection. The theoretical stage aimed to clarify key aspects of the research and detail the instruments used. The practical stage included administering the esthetic component (IOTN-AC) of the IOTN to 15 participants without ASD aged 7 to 14 at a school clinic, along with a dynamic questionnaire. The diagnostic reliability of the IOTN was assessed using the Kappa test, yielding indices of 0.88 (inter-examiner) and 0.91 (intra-examiner). We conducted a pilot study at the rehabilitation center involving 10 individuals with ASD aged 7 to 14 years and their caregivers. The aim was to test the methodological steps of the study. No alterations were made to the proposed methodology for the study. Data obtained in these preliminary phases were not included in the final statistical analysis.

### Data collection

The inclusion criteria for children and adolescents were individuals aged 6 to 14 years with a confirmed diagnosis of ASD in their medical records, as well as children and adolescents without ASD. This age group was selected based on the chronological interval recommended by the assessment instruments used in the study, including the FIS and the IOTN-AC.^17^ Caregivers were required to be literate and proficient in Brazilian Portuguese. We excluded from the sample families of patients with systemic conditions that could affect oral health and patients undergoing or about to start orthodontic treatment.

Family were recruited at the research sites during children/adolescents’ appointments, with all necessary biosafety measures to prevent COVID-19. Parents or guardians responded to three different instruments to assess the impact of the studied conditions on family environment, oral hygiene habits of children/adolescents, and sociodemographic characteristics.

Children and adolescents were assessed for their socio-psychological need for orthodontic treatment. The patients were placed in a secretary chair with a slim leg model, facing forward and smiling, with teeth at maximum habitual intercuspation. In order to reduce measurement biases, we captured photographs for subsequent analysis (DSLR EOS 4000D, Canon®, São Paulo, Brazil) to mitigate potential interference from non-cooperating individuals and ensure greater comparability with evaluations from caregivers. Initially, caregivers and then a professional orthodontist conducted the assessment using the IOTN-AC.

#### Data Source and Variables

The Family Impact Scale (FIS), which consists of 4 domains and 14 items, covering parental/family activities, parental emotions, family conflict, and financial difficulties was used. In this study, our focus was on the parental emotions domain, which included four questions. The questions aim to assess the frequency of events in the last three months, with response options ranging from 0 ("never") to 4 ("almost every day"), including the alternative "don't know." The total score ranges from 0 to 16, with higher values indicating a greater degree of impact of the children/adolescents’ conditions on the caregivers’ and family's emotions as a whole.^18^

The questionnaire on oral hygiene habits inquired about the practices adopted by children/adolescents over the last 12 months through direct questions, including responsibility for tooth brushing, flossing, and tongue brushing. The sociodemographic questionnaire compiled relevant information about the individual and family context. The IOTN-AC was used to assess the socio-psychological need for orthodontic treatment. This subjective component consists of a descending scale of esthetic attractiveness, represented by ten colored photographs. During the assessment, the researcher and caregivers compared the occlusal aspect of the patient with the photograph that showed the closest esthetic resemblance. Each photograph was categorized according to specific treatment needs.^15^ The severity of ASD was confirmed in the patients’ medical records, based on a comprehensive assessment conducted by a multidisciplinary team of healthcare professionals, ranging from mild to moderate to severe.

### Statistical analysis

Parental emotion constituted the study's outcome variable. The independent variables were organized into three hierarchical levels, as required for statistical analysis. This conceptual framework was delineated so that the variables at the top (level 1 or distal) could influence the subsequent ones. According to our hypotheses, the variables with the greatest potential relationship with caregivers were situated at level 3.^19^

The statistical software SPSS version 20.0 (SPSS for Windows, IBM Inc, Armonk, USA) was used for data analysis. Descriptive analyses were conducted, including measures of central tendency and dispersion for quantitative variables, and absolute and relative frequencies for qualitative variables. The normality of the data distribution was assessed using the Kolmogorov-Smirnov test, revealing a non-normal distribution, except for the variable ‘age of caregiver’. The Mann-Whitney test was used to compare quantitative variables between the groups with and without ASD. For the variable ‘age of caregivers,’ the Student's t-test was used. For qualitative variables, the Pearson chi-square test and Fisher's exact test were applied.

The socio-psychological need for orthodontic treatment was assessed based on esthetic perception, according to the classification of photographs from the IOTN-AC. The images were categorized into three groups: "no treatment need" (photos 1 to 4), "moderate treatment need" (photos 5 to 7), and "mandatory treatment need" (photos 8 to 10).^15^

To calculate the effect size of qualitative variables, we used the Phi measure. For the quantitative variable with a normal distribution, we employed Cohen's d. In non-parametric tests, we adopted the bi-serial correlation.

The differences in scores for the ‘parental emotions’ domain and its items between families with and without ASD were compared with the Mann-Whitney test.

Poisson regression with robust variance was used to examine significant associations between independent variables and parental emotion. For this analysis, the outcome variable was categorized as ‘absent’ (corresponding to the response "never" in all questions) and ‘present’ (encompassing the other response categories in at least one question: "Once or twice", "Several times", "Many times", and "Every day"). "Non-responses" were excluded from the analysis. The multivariate analysis model was constructed based on a hierarchical approach, following the theoretical model proposed by Victora et al.^19^. This approach considers three levels of determinants: at Level 1 (distal), variables related to children/adolescents’ oral hygiene habits were included; at Level 2 (intermediate), characteristics of children/adolescents including age, socio-psychological need for orthodontic treatment, and level of ASD were included; and at Level 3 (proximal), socioeconomic aspects of parents/guardians were included. In the bivariate analysis, variables with a p-value < 0.20 were selected for inlcusion in the multivariate model, along with others considered determinants from a theoretical standpoint. In the final regression model, only variables with a p-value < 0.05 in the adjusted analysis were retained. We calculated prevalence ratios (PR) with their respective 95% confidence intervals (CI). A significance level of p < 0.05 was used for all statistical tests.

## Results

This study included 144 children and adolescents and their respective caregivers, divided into two groups of 72 pairs each. Two caregivers did not complete the questionnaire due to scheduling conflicts, and one from the ASD group did not agree with the methodology because the child did not cooperate. Among these caregivers, 78.5% were mothers and 13.9% were fathers. The mean age of caregivers in the ASD group was 34.6 years (SD 6.3), while in the comparison group it was 32.6 years (SD 5.9). Children and adolescents had a mean age of 7.81 years (SD 2.3) in the ASD group and 8.38 years (SD 1.9) in the non-ASD group (p = 0.030), with a predominance of males in public schools. As documented, 51.4% of children and adolescents were diagnosed with mild autism, 38.9% with moderate, and 9.7% with severe. There was a higher number of children per family in the comparison group, with an average of 2.90 (p = 0.049) (Table 1).

**Table 1:** Sample characteristics and comparison between groups with and without ASD.

Quantitative variables		ASD group			Comparison group			p-value	Effect size
		M (SD)	Median (P25; P75)	Range	M (SD)	Median (P25; P75)	Range		
Age of caregivers		34.60 (6.30)	35.00 (29.25; 39.75)	21-49	32.58 (5.88)	33.0 (28.25; 36.0)	20-46	0.049* a	0.33
Age of the child/adolescent		7.81 (2.32)	7.00 (6.00; 9.00)	3/14	8.38 (1.95)	8.00 (7.00; 10.00)	6/13	0.030* b	0.18
Number of children		2.18 (1.08)	2.00 (1.25; 2.75)	1/6	2.90 (1.97)	2.00 (2.00; 375)	1/9	0.047* b	0.17
Qualitative variables				n (%)		n (%)			
Gender of the child/adolescent									
	Male			58 (54.2)		49 (45.8)		0.086^c^	0.14
	Female			14 (37.8)		23 (62.2)			
Person responsible for tooth brushing									
	Patient			19 (22.4)		66 (77.6)		<0.001* c	0.66
	Caregiver			53 (89.8)		6 (10.2)			
Flossing habits									
	Yes			12 (22.2)		42 (77.8)		<0.001* c	0.43
	No			60 (66.7)		30 (33.3)			
Tongue brushing									
	Yes			39 (38.6)		62 (61.4)		<0.001* c	0.35
	No			33 (76.7)		10 (23.3)			
Evaluation of IOTN-AC by the caregiver									
	No need for orthodontic treatment			44 (48.9)		46 (51.1)			
	Moderate need for orthodontic treatment			15 (46.9)		17 (53.1)		0.639^c^	0.079
	Mandatory need for orthodontic treatment			13 (59.1)		9 (40.9)			
Evaluation of IOTN-AC by the professional									
	No need for orthodontic treatment			40 (47.6)		44 (52.4)			
	Moderate need for orthodontic treatment			21 (51.2)		20 (48.8)		0.709^c^	0.069
	Mandatory need for orthodontic treatment			11 (57.9)		8 (42.1)			
Type of school									
	Private			10 (23.8)		32 (76.2)		<0.001* c	0.34
	Public			62 (60.8)		40 (39.2)			
Education level of caregivers									
	Above 11 years			12 (40.0)		18 (60.0)			
	From 9 to 11 years			35 (49.3)		36 (50.7)		0.308^c^	0.13
	Up to 8 years			25 (58.1)		18 (41.9)			
Home situation									
	Owned			40 (44.9)		49 (55.1)		0.123^c^	0.13
	Non-owned			23 (41.8)		32 (58.2)	
Income**									
	≥ 4 minimum wages			2 (22.2)		7 (77.8)			
	From 1 to 3 minimum wages			55 (55.0)		45 (45.0)		0.106^c^	0.18
	Less than 1 minimum wage			15 (42.9)		20 (57.1)			

ASD: Autism Spectrum Disorder; M: mean; P25–P75: interquartile range (IQR); SD: standard deviation; n: absolute frequency;

* p-value less than 0.05;

a Student's t-test;

b Mann–Whitney U test;

c Chi-square test of independence;

** Categories were constructed using the current minimum wage of R$ 1412.

Regarding tooth brushing responsibility, 89.8% of caregivers in the ASD group assumed the task, in contrast to 77.6% in the non-ASD group, where patients were responsible for it themselves (p < 0.001). A lower use of dental floss was observed in the ASD group (p < 0.001). Tongue brushing was also significantly less frequent in this group (p < 0.001). The majority were evaluated by caregivers and specialists as having no or little sociopsychological need for orthodontic treatment.

The majority of caregivers in both groups had 9 to 11 years of education and resided in their own homes, with income ranging from 1 to 3 minimum wages. Sample characteristics are detailed in Table 1.

The total score for parental emotions was significantly higher in families of children and adolescents with ASD (p < 0.001). Except for the perception of family guilt, the other questions had significantly higher ratings by caregivers in the ASD group (Table 2).

**Table 2:** Comparative analysis of total parental emotion scores and their items.

Variables		ASD group		Comparison group		p-value	Effect size
		M (SD)	Median (P25; P75)	M (SD)	Median (P25; P75)		
Total score of parental emotions		5.64 (3.69)	6.00 (3.00; 8.00)	2.10 (2.51)	2.00 (0; 3.00)	< 0.001	0.49
Domain questions							
	Family disruption	1.35 (1.32)	1.00 (0; 3.00)	0.65 (1.15)	0 (0; 1.00)	< 0.001*	0.30
	Family guilt feeling	0.65 (1.05)	0 (0; 1.00)	0.44 (0.82)	0 (0; 0.70)	0.247	0.10
	Concern about the child's future opportunities	2.04 (1.58)	3 (0; 3.00)	0.72 (1.12)	0 (0; 2.00)	< 0.001*	0.44
Discomfort in public places with the child		1.60 (1.32)	2.00 (0; 3.00)	0.26 (0.61)	0 (0; 0)	< 0.001*	0.53

ASD: Autism Spectrum Disorder; M: mean; P25–P75: interquartile range (IQR); SD: standard deviation;

* p-value less than 0.05.

The regression model was used due to the high frequency of the outcome in this sample (75.7%; n = 109), in which parents reported experiencing some level of emotions associated with the assessed conditions. In the bivariate analysis, an association was observed between parental emotions and the following independent variables: caregivers’ age, responsibility for brushing, dental floss use, tongue brushing, IOTN-AC assessment by caregivers and professionals, ASD severity, type of school attended, caregivers’ education, housing conditions, and income level. In the final adjusted model, the following variables remained significant: responsibility for brushing, IOTN-AC assessment by caregivers, caregivers’ education, and family income (Table 3).

**Table 3:** Hierarchical Poisson regression analysis of Parental Emotions and independent variables in families with and without ASD.

Variables			Unadjusted PR (95% CI)		Adjusted PR (95% CI)	
			95%CI	p–value	95%CI	p–value
Age of caregivers			1.014 (1.000–1.028)	0.042*	–	–
Level 1						
	Person responsible for brushing					
		Patient	1.00		1.00	
		Caregiver	1.363 (1.144–1.625)	0.001*	1.336 (1.121–1.591)	0.001**
	Flossing habits					
		Yes	1		–	–
		No	1.324 (1.057–1.657)	0.014*	–	–
	Tongue brushing					
		Yes	1		–	–
		No	1.257 (1.064–1.485)	0.007*	–	–
Level 2						
	Age of the child/adolescent		0.996 (0.954–1.041)	0.868	–	–
	Evaluation of IOTN-AC by the caregiver					
		No need for orthodontic treatment	1		1	
		Moderate need for orthodontic treatment	0.938 (0.719–1.222)	0.633	0.918 (0.710–1.187)	0.515
		Mandatory need for orthodontic treatment	1.302 (1.115–1.519)	0.001*	1.248 (1.069–1.457)	0.005**
	Evaluation of IOTN-AC by the professional					
		No need for orthodontic treatment	1		–	–
	Moderate need for orthodontic treatment		1.075 (0.872–1.324)	0.498	–	–
		Mandatory need for orthodontic treatment	1.160 (0.917–1.467)	0.216*	–	–
	Severity of ASD					
		Mild	1		–	–
		Moderate	1.115 (0.964–1.290)	0.144*	–	–
		Severe	1.156 (1.018–1.313)	0.025*	–	–
	Type of school					
		Private	1		–	–
		Public	1.384 (1.061–1.803)	0.016*	–	–
Level 3						
	Education level of caregivers					
		Above 11 years	1		1	
		From 9 to 11 years	1.452 (1.016–2.077)	0.041*	1.424 (0.980–2.070)	0.064
		Up to 8 years	1.657 (1.165–2.356)	0.005*	1.454 (1.023–2.066)	0.037**
		Number of children	1.010 (0.952–1.072)	0.735	–	–
	Home situation					
		Owned	1		–	–
		Non-owned	0.866 (0.705–1.064)	0.170*	–	–
	Income					
		≥ 4 minimum wages	1		1	
		From 1 to 3 minimum wages	0.844 (0.652–1.091)	0.195*	0.584 (0.369–0.925)	0.022**
		Less than 1 minimum wage	0.836 (0.618–1.131)	0.244	0.572 (0.352–0.929)	0.024**

PR: Prevalence ratio; CI: confidence interval; IOTN-AC: Aesthetic Component of the Index of Orthodontic Treatment Need; ASD: Autism Spectrum Disorder;

* Variables included in the multivariate model (p < 0.20);

** Variables that remained in the final multivariate model (p < 0.05).

## Discussion

A high prevalence of self-reported emotions was found among caregivers of individuals with and without ASD. Aspects related to parental education, socio-psychological need for orthodontic treatment, and daily caregiving responsibilities for children and adolescents influenced this finding. This study is likely the first one to investigate parental emotions in families with and without ASD using psychometrically robust instrument .^18^

In the multiple regression analysis, caregiver age was significantly associated with individuals’ emotions, indicating that older caregivers tend to experience more emotions. These findings are consistent with previous results that indicated that older mothers had greater concerns about their children's oral health.^16^ Previous data corroborate the link between older age and reduced quality of life in caregivers of people with disabilities, but caution is needed when inferring associations as the mean age of the participants in that study was 78.1 years.^20^ The accumulation of responsibilities related to individuals with ASD is likely to trigger emotions in caregivers, and concerns about their children's adulthood may contribute to the intensification of these emotions.

In our study, caregivers of children and adolescents with ASD reported significantly higher emotional scores, for both the total score and the domain questions, with the exception of family guilt, in line with previous investigations.^11,21^ Previous studies have reported a decline in the quality of life of parents of children with ASD, as well as significantly higher parental emotions in families with ASD.^6,16^ This demonstrates that concerns about the impact of ASD on parental/caregiver emotions are also reflected in the perception of OHRQoL. Thus, parental emotions should not only be discussed within the academic community, but also at the governmental level, aimed at formulating public policies targeting families of individuals with disabilities.

The elevated emotional scores observed in caregivers of individuals with ASD are due to a complex interplay of factors, encompassing the characteristics of the child/adolescent, their level of dependence, parental caregiving styles, and challenges associated with accessing oral and general health care. Concerns regarding the behavior of individuals with ASD, including emotional dysregulation and externalizing behaviors, significantly correlate with increased emotional tension experienced by the majority of caregivers.^22^ Furthermore, challenges in managing restricted and repetitive behaviors, as well as greater dependence for basic daily activities, exacerbate caregiver's burden^3^. This burden is further compounded by the heightened risk of both oral and general health problems.^11,21^ Another probable set of predictors stems from the challenge of balancing work routines with caring for individuals with autism or even experiencing unemployment. Additionally, a more permissive and less authoritarian parenting style was frequently observed in families with ASD and may be adversely affected by parental anxiety.^23^ The majority of individuals with ASD have difficulty accessing dental health services, which undermines comprehensive care.^24^ Parents who face barriers in accessing these services may experience feelings of anguish, irritability, and frustration, thereby affecting their emotional well-being.

In our sample, we found no significant difference in parental guilt between the groups with and without ASD. The analysis should be thorough and can be guided by various aspects, especially considering the breadth of this concept, which include objective aspects such as doing or having done something harmful and responsibility, as well as subjective aspects such as remorse, self-blame, and personal failure. In a study addressing self-forgiveness, guilt, shame, and parental stress in parents of children with ASD and parents of neurotypical children, parental stress showed a strong correlation with guilt and shame.^25^ The variability in the interpretation of guilt associated with the shame of parenting an autistic child may explain the absence of a significant difference between the groups in our study regarding this question.

Engaging in collective events, although generally beneficial for health, is experienced differently by caregivers of children with ASD. Mothers tend to spend more time caring for their children than actively participating in recreational activities.^26^ In the present study, caregivers of individuals with ASD reported greater discomfort in public settings compared to neurotypical families. One scoping review revealed that mothers of children with ASD face emotional challenges during social events, such as fear, stress, and anxiety, influenced by factors such as social stigma, lack of support, and unpredictable behaviors of autistic children.^27^ Furthermore, other data have shown that caregivers face challenges related to social reactions and feelings of embarrassment within the family environment.^28,29^ To mitigate this impact, strategies such as occupational therapy,^27^ psychological support, and social support^30^ are valid, as the regularity and effectiveness of support for ASD is associated with a positive impact on emotional well-being.

In our sample, 89.8% of caregivers were responsible for brushing the teeth of children/adolescents with ASD. The adjusted multivariate model revealed that the caregiver's responsibility for brushing increases the prevalence of parental emotions by 33.6% compared to situations where the child/adolescent takes on this responsibility themselves. Similar findings were observed in other studies;^21,31^ however, our study indicated an even higher frequency of parents assuming this responsibility in the ASD group. This occurs because children with ASD need assistance with oral hygiene and sometimes refuse or do not cooperate, making it difficult to maintain oral health.^21^ Furthermore, the increased responsibility assigned to caregivers, the decreased manual dexterity and higher oral sensitivity of some individuals with autism, the fear that their child develops dental caries, and the accumulation of daily tasks can increase the emotional burden of these caregivers. A multidisciplinary support approach for these caregivers is essential, particularly to raise awareness about the importance of oral hygiene and to provide them with oral hygiene techniques. Our study also showed that flossing and tongue brushing yielded significantly more unfavorable outcomes in children with ASD, corroborating previous findings^11,21,31^ and reinforcing the hypotheses discussed in this study.

Previous research has evaluated the IOTN-AC in individuals with ASD,^11,14,32^ but further investigations are needed, particularly regarding its impact on the QoL of caregivers.^32^ Our data indicated that there was no significant difference in the socio-psychological need for orthodontic treatment among children and adolescents with and without ASD. Similar findings were found in a study that used the Dental Aesthetic Index (DAI),^33^ which prioritizes esthetic characteristics and is similar to the AC component of the IOTN. In the multivariate analysis, our results indicated that a higher socio-psychological need for orthodontic treatment, as assessed by caregivers, increases the prevalence of their emotions by 24.8%. This association can be attributed to the distress felt by caregivers regarding the cost of orthodontic treatment, the difficulty in finding orthodontists trained to treat children with ASD, and the challenge of adapting these children to treatments. Additionally, the abandonment of potentially deleterious oral habits should be highlighted, considering that some patients are more prone to develop them.^14^ Based on our data, it is important to note the socio-psychological impact that the esthetic burden of malocclusion have on caregivers. Our findings reinforce the importance of the esthetic component in caregivers’ health decision-making, which tends to focus more on subjective characteristics than on occlusal ones.

The relationship between sociodemographic characteristics and multiple domains of family QoL has not been studied in depth.^34^ Our results indicate an association between lower caregiver education levels and a higher likelihood of reporting parental emotions, corroborating previous findings.^21^ Our findings may be explained by several hypotheses. Firstly, caregivers with lower levels of education may have less knowledge about oral health, oral hygiene, and how to handle adverse oral health-related situations. Secondly, other health issues that overlap with oral health problems may mask this relationship. Additionally, individuals with low levels of education may be less likely to have high incomes, which can be a concern, especially in the context of the pandemic, resulting in a high unemployment rate.^7^

Interestingly, our data indicated that the likelihood of experiencing parental emotions decreased as the reported income decreased, contrary to other studies.^21,30^ Other variables not addressed in this study may influence this association. Social support and access to welfare services deserve attention.^30^ Additionally, social assistance programs may have influenced the result, as some participants may not consider these benefits as official income. Other caregivers may have concealed their true financial condition for fear of losing government benefits. Thus, future research addressing this information is encouraged.

The current study acknowledges the limitations of the cross-sectional design, which preclude causal inferences. Therefore, longitudinal studies are essential for further investigation, addressing other age groups of patients with ASD. Moreover, memory bias in questionnaires can be a concern. Despite the limitations, our study presents positive aspects. It includes the use of validated questionnaires, encompassing both objective burden, related to the practical challenges of parenting, and subjective burden, concerning caregivers’ psychological reactions as measured by the FIS domain, thus addressing identified gaps.^9^ Furthermore, the present investigation provides valuable insights into the topic, and recognition of the associated factors may enable the development of strategies to prevent or minimize the effects related to parental emotions. The importance of early orthodontic intervention is crucial in preventing more invasive procedures in the future, thus reducing costs and financial impact on families. Our research has shown that families with ASD face greater emotional burdens, highlighting the need for recognition and support from healthcare professionals and policy makers.^6^

## Conclusion

Our findings unveiled a notably higher prevalence of parental emotions within the group of families with ASD. Parental emotions were associated with caregiver responsibility for tooth brushing of children and adolescents, caregivers’ perception of greater socio-psychological need for orthodontic treatment, and caregivers’ lower education level. Income was found to be as a protective factor for parental emotions.
